# Glucose Oxidase Induces Cellular Senescence in Immortal Renal Cells through ILK by Downregulating *Klotho* Gene Expression

**DOI:** 10.1155/2015/416738

**Published:** 2015-10-25

**Authors:** Nuria Troyano-Suárez, María del Nogal-Avila, Inés Mora, Patricia Sosa, Susana López-Ongil, Diego Rodriguez-Puyol, Gemma Olmos, María Piedad Ruíz-Torres

**Affiliations:** ^1^Departamento de Biología de Sistemas, Universidad de Alcalá, Alcalá de Henares, 28871 Madrid, Spain; ^2^University of Alabama at Birmingham, Birmingham, AL, USA; ^3^Unidad de Investigación, Fundación para la Investigación Biomédica del Hospital Universitario Príncipe de Asturias, Alcalá de Henares, 28871 Madrid, Spain; Instituto Reina Sofía de Investigación Nefrológica, IRSIN, Madrid, Spain ^4^ Instituto Reina Sofía de Investigación Nefrológica, IRSIN, Madrid, Spain

## Abstract

Cellular senescence can be prematurely induced by oxidative stress involved in aging. In this work, we were searching for novel intermediaries in oxidative stress-induced senescence, focusing our interest on integrin-linked kinase (ILK), a scaffold protein at cell-extracellular matrix (ECM) adhesion sites, and on the *Klotho* gene. Cultured renal cells were treated with glucose oxidase (GOx) for long time periods. GOx induced senescence, increasing senescence associated *β*-galactosidase activity and the expression of p16. In parallel, GOx increased ILK protein expression and activity. Ectopic overexpression of ILK in cells increased p16 expression, even in the absence of GOx, whereas downregulation of ILK inhibited the increase in p16 due to oxidative stress. Additionally, GOx reduced *Klotho* gene expression and cells overexpressing Klotho protein did not undergo senescence after GOx addition. We demonstrated a direct link between ILK and *Klotho* since silencing ILK expression in cells and mice increases *Klotho* expression and reduces p53 and p16 expression in renal cortex. In conclusion, oxidative stress induces cellular senescence in kidney cells by increasing ILK protein expression and activity, which in turn reduces *Klotho* expression. We hereby present ILK as a novel downregulator of *Klotho* gene expression.

## 1. Introduction

Cellular senescence is a permanent cell cycle arrest accompanied by the alteration of the cell structure and functions. Senescence can be promoted in response to stress stimuli that result in DNA damage or by the replicative life of cells. Senescence induction can have beneficial effects in pathologies such as cancer or wound healing; however, the permanent presence of senescent cells in several tissues can induce or increase some pathologies [1]. Cellular senescence was discovered by Hayflick and Moorhead [2], whose experiments showed the limited number of cell divisions in cultured cells. This limited proliferation of cells is promoted by a shortening of telomeres in a chromosome [3], which, if too short, can induce cell senescence [4]. Moreover, cell senescence can be induced by other telomere-independent mechanisms, such as oncogene activation [5], DNA damage [6], or stressful stimuli called stress-induced premature senescence [7, 8]. Recent studies from our group have demonstrated that hyperosmolar stress induced by high glucose concentration and Amadori products promoted premature senescence in kidney cells [9, 10].

Senescent cells are not able to proliferate and present some morphological and biochemical changes, such as increased activity of *β*-galactosidase (SA-*β*-GAL) due to an increase in lysosomal content [11] and in the expression of cell cycle inhibitors such as p16 or p53 tumor suppressor genes.

Oxidative stress is involved in many diseases, such as cancer and inflammation, and it plays a relevant role in aging [12–14]. The evidence that reactive oxygen species (ROS) are involved in the senescence process has been provided by the use of antioxidant compounds that can prevent or delay cellular senescence [15]. ROS such as H_2_O_2_ or superoxide anions produce acute damage to proteins, lipids, and DNA. In addition, some works have shown that they can induce aging by the activation of senescence genes through MAPK cascade and their downstream kinase effector p38 [16, 17] and replicative senescence [14]. Thus, it has been shown that ROS can downregulate Klotho [18, 19], an aging-related kidney-secreted hormone with antioxidant properties [20]. Klotho is a glucuronidase activity protein which acts as a coreceptor for fibroblast growth factor 23 (FGF23) and regulates phosphate homeostasis and IGF-1 signaling [21–23]. Klotho is downregulated in chronic kidney diseases (CKD) where aging features are accelerated. In fact, Klotho-deficient mice show multiple age-related pathologies and a short lifespan.

In the last years, the integrin-linked kinase protein (ILK) has emerged with a relevant role in kidney and vascular physiology and physiopathology, mediating a relationship between extracellular matrix (ECM) and intracellular processes. ILK is a protein which is part of a cytoplasmic multiprotein complex and mediates the interaction between ECM proteins and intracellular pathways by a serine-threonine kinase activity [24]. This interaction between ECM proteins and the integrin receptor activates ILK, which regulates cellular processes such as proliferation, survival, migration, and fibrosis [25, 26]. However, only few works have established a relationship between ILK and the senescence process [27, 28] and its role in the aging process is still unknown.

In the present work, we test the hypothesis that ROS induce premature cellular senescence in renal cells through the increase in ILK expression and activity and the downregulation of Klotho protein expression, which modulate the expression of senescence genes such as p16.

## 2. Material and Methods

### 2.1. Reagents

Culture plates, culture media, blueStar-prestained protein marker, BCA protein assay reagent, CL-Xposure films, and SuperSignal West Pico detection system were from Cultek (Thermo Fisher Scientific, Madrid, Spain). The secondary horseradish peroxidase-conjugated goat anti-mouse IgG was from Dako Cytomation (Glostrup, Denmark). The secondary horseradish peroxidase-conjugated goat anti-rabbit IgG was from Merck Millipore (Darmstadt, Germany). Acrylamide-bisacrylamide was from Hispanlab-Pronadisa (Madrid, Spain). Electrophoresis equipment and PVDF membrane were from Bio-Rad Laboratories (Richmond, CA, USA). Protease inhibitor cocktail tablets were from Roche (Mannheim, Germany). The *β*-galactosidase substrate (C12FDG, 5-dodecanoyl-aminofluorescein di-*β*-D-galactopyranoside), the mounting medium ProLong Gold antifade reagent with DAPI, Trizol reagent, OptiMEM medium, Lipofectamine, ILK siRNA, and negative control used as a nonsilencing control were from Life Technologies (Paisley, UK). Polyclonal rabbit anti-p53 and monoclonal rabbit anti-p16 were from Abcam (Cambridge, UK). Polyclonal rabbit anti-ILK1, monoclonal rabbit antibodies for anti-phospho-GSK-3*β* (Ser9) and anti-GSK-3*β*, and the buffer kinase 10X, ATP, and GSK-3*β* fusion protein were from Cell Signaling Technology Inc. (Boston, MA, USA). Polyclonal rabbit anti-4-hydroxy-2-nonenal adducts were from Alexis (Farmingdale, NY, USA). Glucose oxidase, bovine serum albumin (BSA), polyclonal rabbit anti-actin, and monoclonal mouse anti-GAPDH were from Sigma-Aldrich-Fluka Chemical Co. (St. Louis, MO, USA).

### 2.2. Culture Cells

Immortal mouse cortical tubule (MCT) cells are a cultured line of proximal tubular cells harvested originally from the renal cortex of SJL mice [29]. The cells were maintained in culture in DMEM supplemented with penicillin 100 U/mL and streptomycin 100 *μ*g/mL and 10% heat-inactivated fetal bovine serum (FBS) in an atmosphere of 95% air and 5% CO_2_ at 37°C, as previously described [29, 30]. Embryonic kidney epithelial human cells, HEK293T cells, were purchased from American Type Culture Collection (Rockville, MD, USA) and they were grown in Eagle's Minimum Essential Medium supplemented with 10% FBS. Culture media were changed every 2 days. For the experiments, both types of cells were treated with 2.5 mU/mL glucose oxidase (GOx) at different times.

### 2.3. Protein Extraction and Immunoblot Analysis

Total protein extracts from cultured cells were obtained by using the Lysis Buffer (20 mM Tris-HCl pH 7.5, 150 mM NaCl, 1 mM EGTA, 1 mM EDTA, 0.1% sodium deoxycholate, 1% Triton X-100, and 10 mM sodium pyrophosphate) containing a protease inhibitor cocktail. The resulting solution was spun at 13,000 rpm for 30 min at 4°C. The protein concentration was determined by BCA protein assay. Equal amounts of protein (30 *μ*g protein/lane) from each sample were separated on SDS-polyacrylamide gels under reducing conditions and transferred onto PVDF membranes. Membranes were blocked with 5% nonfat dry milk or 5% BSA in Tween Tris buffered saline (TTBS) (20 mM Tris–HCl pH 7.5, 0.9% NaCl, and 0.05% Tween 20) for 1 h at room temperature and then incubated overnight at 4°C with different specific antibodies for mouse (anti-p53, anti-p16, anti-ILK1, anti-phospho-GSK-3*β* (Ser9), and anti-GSK-3*β*). Phospho-GSK-3*β* and GSK-3*β* were used to measure ILK activity. After washing in TTBS, the membranes were incubated with horseradish peroxidase-conjugated goat anti-mouse IgG or goat anti-rabbit IgG as secondary antibodies. The immunoreactive bands were visualized with the SuperSignal West Pico detection system after 30 sec of exposure to CL-Xposure films. Then blots were reblotted with a rabbit anti-actin antibody in order to normalize p53, p16, or ILK1 levels.

Proteins from kidney cortex portions were obtained using the Lysis Buffer and, after incubation on ice for 30 min, tissues were homogenized and spun at 13,000 rpm for 30 min at 4°C.

### 2.4. Detection of Senescence Associated *β*-Galactosidase Activity by Fluorescence Confocal Microscopy

To determine cellular senescence, SA-*β*-GAL activity was measured by fluorescence confocal microscopy, using the fluorogenic substrate C12FDG [11, 31]. MCT cells grown in microscope cover glasses were treated with 33 *μ*M C12FDG for 4 h. At the end of incubation, cells were washed twice with PBS and fixed with 4% paraformaldehyde for 15 min. Subsequently, cells were washed again and mounted in ProLong Gold antifade reagent with DAPI overnight. Samples were analyzed using LEICA TCS-SP5 confocal microscope (Leica Microsystems; Wetzlar, Germany) at 488 nm argon laser to detect the fluorescence of SA-*β*-GAL activity and at 405 nm to detect DAPI. Pictures were obtained and fluorescence intensity was measured by densitometry by Image J software (http://rsbweb.nih.gov/ij/).

### 2.5. Transfection of ILK and Klotho

Subconfluent MCT cells (60% to 80% confluent) cultured in 6-well plate were transfected with plasmids containing ILK or Klotho.

For transient transfection of ILK [32], cells were transfected with different doses of a plasmid containing ILK wild type (0, 1, and 2 *μ*g) (ILK-WT which was provided by Dr. S. Dedhar, University of British Columbia, Canada). Then, each one was mixed with Lipofectamine (5 *μ*L/well) and incubated for 15 minutes at room temperature for 24 hours prior to addition to the cells.

For stable transfection of Klotho [10, 33], cells were transfected with 1 *μ*g of a plasmid containing the transmembrane form of mouse Klotho cloned into pEF1/Myc-His vector (pEF1-Klotho) or with the empty pEF1/Myc-His vector as control (pEF1-Empty). Both plasmids were kindly provided by Dr. M. Kuro-O from the University of Texas Southwestern Medical Center, Texas. For transfection, Opti-MEM media and Lipofectamine were used. To select the transfected MCT cells, cells were treated with 200 *μ*g/mL of G418 (Invitrogen) for 14 days. Transfection of Klotho in HEK293T cells was transient as these cells have resistance to Geneticin and they cannot be selected with G418.

At the end of transfection, media were removed and replaced with complete media for 24 h, after which MCT cells were lysed. Protein concentration was measured to analyze p16 protein levels by western blot, and RNA was isolated to test* Klotho* mRNA levels.

### 2.6. Measurement of mRNA Expression

The total RNA from MCT cells or kidney cortex from mice was isolated using Trizol reagents according to the manufacturer's protocol. The RNA integrity was checked using agarose-formaldehyde gels, and the RNA concentration was measured using a Vis-UV spectrophotometer (Nanodrop). cDNA was synthesized using a high capacity cDNA reverse transcription kit (Applied Biosystems Inc., Foster City, CA, USA), and* Klotho*, ILK-1, and GAPDH expression was measured by quantitative RT-PCR (qPCR) (ABI Prism 7000), using Taqman genes and double delta Ct method. Klotho (Mm00473122_m1), ILK-1 (Mm01274251_g1), and the endogenous control GAPDH (Mm99999915_g1) were used (Applied Biosystems Inc., Foster City, CA, USA).

### 2.7. ILK siRNA Transfection

ILK was silenced in MCT cells by transfecting a specific small interfering RNA against ILK (siILK). An unspecific scrambled RNA was used as transfection control (scRNA). Both siILK and scRNA were transfected using Lipofectamine 2000 for 24 h. Cells were incubated with complete RPMI for 24 h. Cells were processed in duplicate to evaluate p16 expression by western blot after adding GOx for 48 h using serum-free RPMI and to check ILK and* Klotho* mRNA expression by RT-qPCR, as described above.

### 2.8. Conditional ILK Knockout Mouse Model

All animal procedures were in accordance with the EU Directive 2010/63/EU and they were previously approved by the Institutional Animal Care and Use Committee at the University of Alcala. Animals were housed in a pathogen-free and temperature-controlled room (22 ± 2°C). Food and water were available ad libitum. Conditional inactivation of the ILK gene was accomplished by crossing C57Bl/6 mice homozygous for the floxed ILK allele, flanked by loxP sites (ILKfl/fl), with homozygous mice carrying a tamoxifen-inducible CreER (T) recombinase gene (CRE^+/+^) which express Cre under the control of the cytomegalovirus promoter [34, 35]. Eight-week-old male mice were injected intraperitoneally with 1.5 mg of tamoxifen (TX) (Sigma Co., St. Louis, MO, USA) (dissolved in a 10 : 1 volumetric mix of corn oil and ethanol) or Vehicle alone (VH), once a day for 5 consecutive days to induce ILK deletion. After 5, 15, and 30 days following VH or TX injections, routine genotyping of tail DNA samples was performed to monitor the Cre-driven ILK deletion [36, 37]. TX-treated CRE-LOX mice displaying successful deletion of ILK are named conditional KO-ILK (cKO-ILK) mice and their control VH-treated CRE-LOX are named wild type (WT). Animals were euthanized 20 days after the last injection.

The p16 and p53 expression in renal cortex from WT or cKO-ILK mice was assessed by western blot as well as ILK and* Klotho* mRNA levels by RT-qPCR in these samples, as described above.

### 2.9. Statistical Analysis

Results are shown as mean ± standard error of the mean (s.e.m.) of a variable number of experiments, detailed in figure captions. Most experiments are presented as fold increase in basal or control values. To compare the different experimental situations, 1-way or 2-way ANOVA was used, depending on the experiments. Pairwise comparisons were performed using Fisher's least significant difference method. The Dunnett test was used to analyze the changes in respect to basal values. All tests were two-tailed, and a value of *p* < 0.5 was considered statistically significant.

## 3. Results

### 3.1. Glucose Oxidase Induced Cellular Senescence in Renal Mouse Cortical Tubule (MCT) Cells Increasing the ILK Expression and Activity

**Figure 1:**
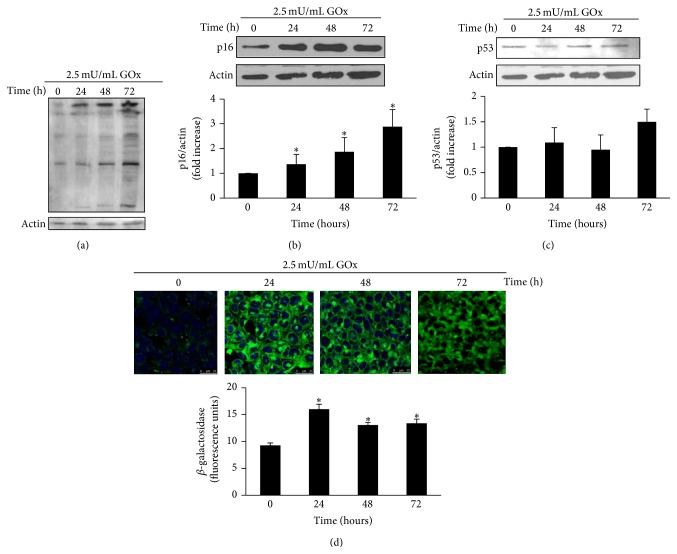
*GOx induces senescence in wild-type mouse cortical tubule (MCT) cells*. MCT cells were treated with 2.5 mU/mL GOx for 24, 48, or 72 h. (a) 4-Hydroxy-2-nonenal proteins adducts were analyzed by western blot. (b) p16 and (c) p53 protein expressions were analyzed by western blot. A representative blot is shown in each case. Bar graphs represent the densitometric analysis of the bands. The results are expressed as densitometric units and are the mean ± s.e.m. from six different experiments. (d) SA-*β*-GAL activity was analyzed by confocal microscopy using the fluorescent substrate C12FDG. A representative experiment is shown. Bar graph represents the analysis of the fluorescence from five different experiments and the mean ± s.e.m. is expressed as fluorescence units. ^*∗*^ *p* < 0.05 versus control (time 0).

MCT cells were treated with 2.5 mU/mL GOx for 24, 48, and 72 h, to induce oxidant stress. GOx induced a time-dependent increase in oxidative damage, which was evaluated quantifying the formation of 4-hydroxynonenal protein adducts by western blot (Figure 1(a)). Protein expression of senescence genes was evaluated by western blot. Results showed a time-dependent increase in p16 expression (Figure 1(b)), whereas no significant changes were found in p53 expression (Figure 1(c)). GOx also increased SA-*β*-GAL activity detected with confocal microscopy, indicating an increase in the percentage of senescent cells 24 h after GOx treatment (Figure 1(d)).

Next, to analyze whether there was a link between the increase in ILK and the induction of senescence, we overexpressed ILK on MCT cells by transfection with a plasmid containing a wild-type ILK protein (WT-ILK).  Figure 3(a) shows how the expression of ILK increases in MCT cells transfected with WT-ILK in a dose-dependent way. MCT cells overexpressing ILK also had a strong expression of senescence gene p16, even in the absence of oxidant stress (Figure 3(a)). Moreover, ILK expression was knocked down by transfecting cells with specific siILK and then treated with 2.5 mU/mL GOx for 48 h. Transfection significantly reduced ILK mRNA expression, measured by RT-qPCR (Figure 3(b)), and also the protein content, measured by western blot (Figure 3(c)). Cells transfected with siILK did not show the expected increase in p16 expression after GOx treatment compared to cells transfected with scRNA (Figure 3(c)). Both results allow establishing a direct link between the increase in ILK protein content and increased senescence gene p16 expression.

**Figure 2:**
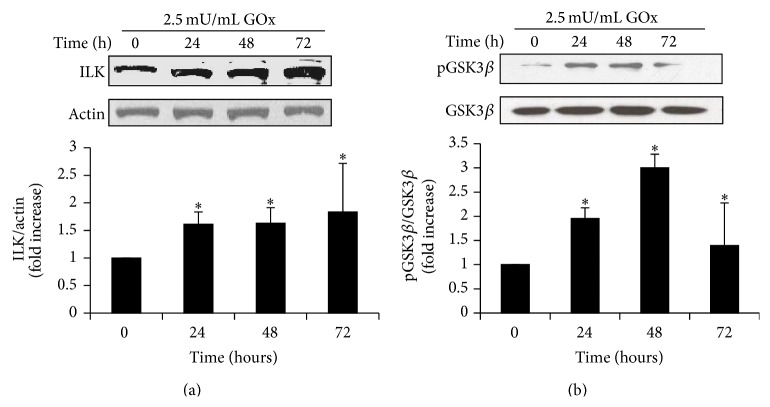
*GOx induces an increase in ILK protein expression and activity*. MCT cells were treated with 2.5 mU/mL GOx for 24, 48, or 72 h. (a) ILK and (b) phospho-GSK3*β* and GSK3-*β* protein expression was analyzed by western blot. A representative blot is shown in each case. Bar graphs represent the densitometric analysis of the bands. The results are expressed as densitometric units and are the mean ± s.e.m. from four different experiments. ^*∗*^ *p* < 0.05 versus control (time 0).

**Figure 3:**
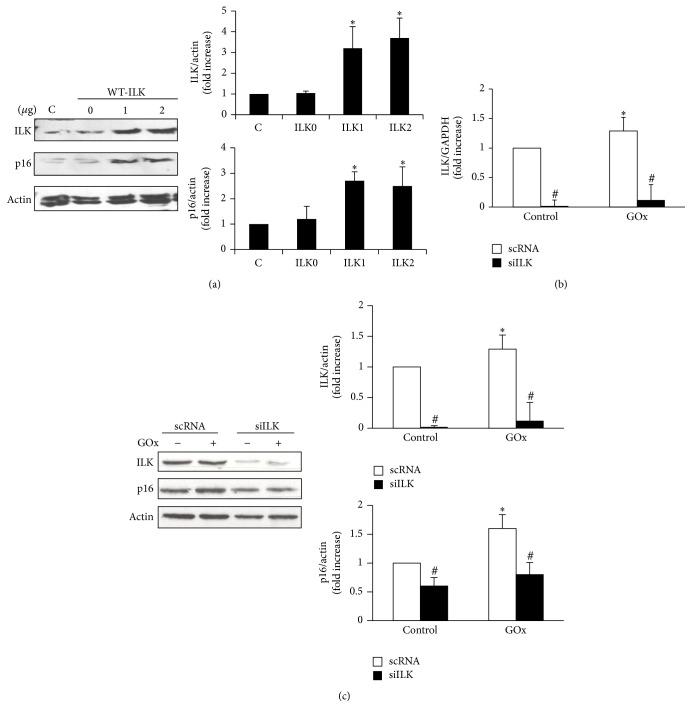
*ILK mediates cellular senescence induced by GOx*. (a) MCT cells were transfected with a plasmid coding for wild-type ILK protein (WT-ILK) at several doses (0, 1, or 2 *μ*g). ILK and p16 proteins were evaluated by western blot. (b, c) MCT were transfected with the specific siRNA for ILK (siILK) or unspecific scramble siRNA (scRNA) as control and then treated with 2.5 mU/mL of GOx for 48 h. In those conditions, mRNA ILK was evaluated by RT and real time PCR (b) and ILK and p16 protein expression was evaluated by western blot (c). A representative blot is shown in each case. Bar graphs represent the densitometric analysis of the bands. The results are expressed as densitometric units and are the mean ± s.e.m. of the mean from five different experiments. ^*∗*^ *p* < 0.05 versus control; ^#^ *p* < 0.05 versus scRNA.

In parallel, we analyzed the effect of oxidant stress on ILK expression and activity and found that GOx induced a significant increase in the protein expression at all times examined (Figure 2(a)). GOx also induced an increase in the ILK activity evaluated as the phosphorylation of its substrate GSK-3*β*, as shown in Figure 2(b), without changes in the protein content of total GSK-3*β*.

### 3.2. Glucose Oxidase Reduced the Expression of the Gene* Klotho* in MCT Cells

**Figure 4:**
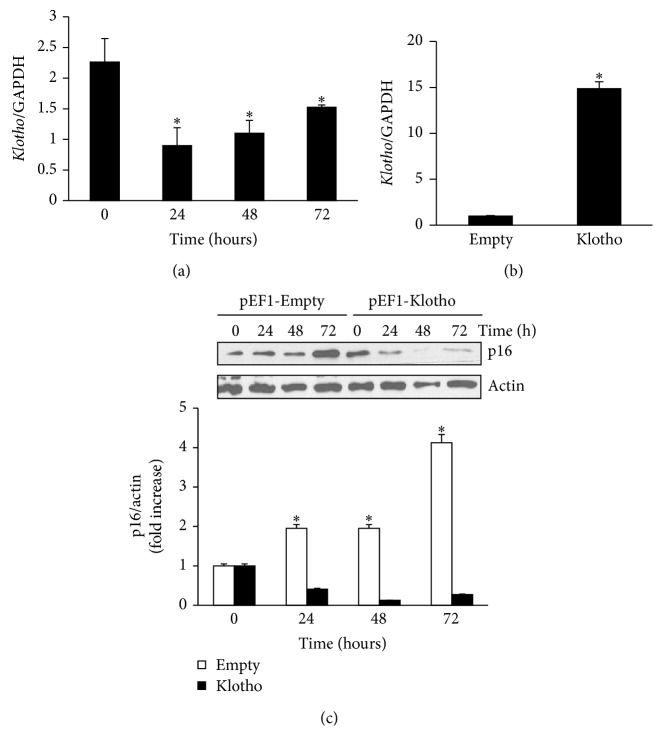
*Antiaging gene Klotho is modulated by GOx and mediates cellular senescence induced by GOx*. (a) MCT cells were treated with 2.5 mU/mL of GOx for 24, 48, and 72 h and mRNA* Klotho* expression was analyzed by RT and real time PCR. Bar graph represents the mean ± s.e.m. from three different experiments. MCT cells were transfected with a plasmid containing Klotho protein (pEF1-Klotho) or with an empty vector (pEF1-Empty) as control, and (b) mRNA* Klotho* expression was analyzed by RT and real time PCR. Bar graph represents the mean ± s.e.m. of three different experiments. (c) MTC cells transfected with Klotho protein were treated with 2.5 mU/mL of GOx for 24, 48, and 72 h and p16 protein expression was evaluated by western blot. A representative blot is shown. Bar graphs represent the densitometric analysis of the bands. The results are expressed as densitometric units and are the mean ± s.e.m. from five different experiments. ^*∗*^ *p* < 0.05 versus control (time 0).

MCT cells were treated with 2.5 mU/mL GOx for 24, 48, and 72 h, and* Klotho* mRNA expression was analyzed by RT-real time PCR. We found that oxidative stress significantly reduced the mRNA expression of* Klotho* gene 24 h after GOx addition (Figure 4(a)). To analyze whether the reduction in* Klotho* expression could be related to the increase in senescence gene p16, MCT cells were transfected with a plasmid containing Klotho protein. The transfection of pEF1-Klotho induced a strong increment in* Klotho* mRNA expression compared with transfection of pEF1-Empty (Figure 4(b)). The highest expression of Klotho remained until 72 h after transfection (data not shown).

After transfection, cells were treated with 2.5 mU/mL GOx and p16 expression was evaluated by western blot. Cells transfected with the empty vector (pEF1-Empty) responded to GOx treatment increasing p16 expression, as expected, whereas cells overexpressing Klotho protein (pEF-1-Klotho) did not increase the expression of senescence gene p16 (Figure 4(c)).

### 3.3. Reduced* Klotho* Expression Depended on the Increase in ILK Expression in Response to Oxidative Stress

**Figure 5:**
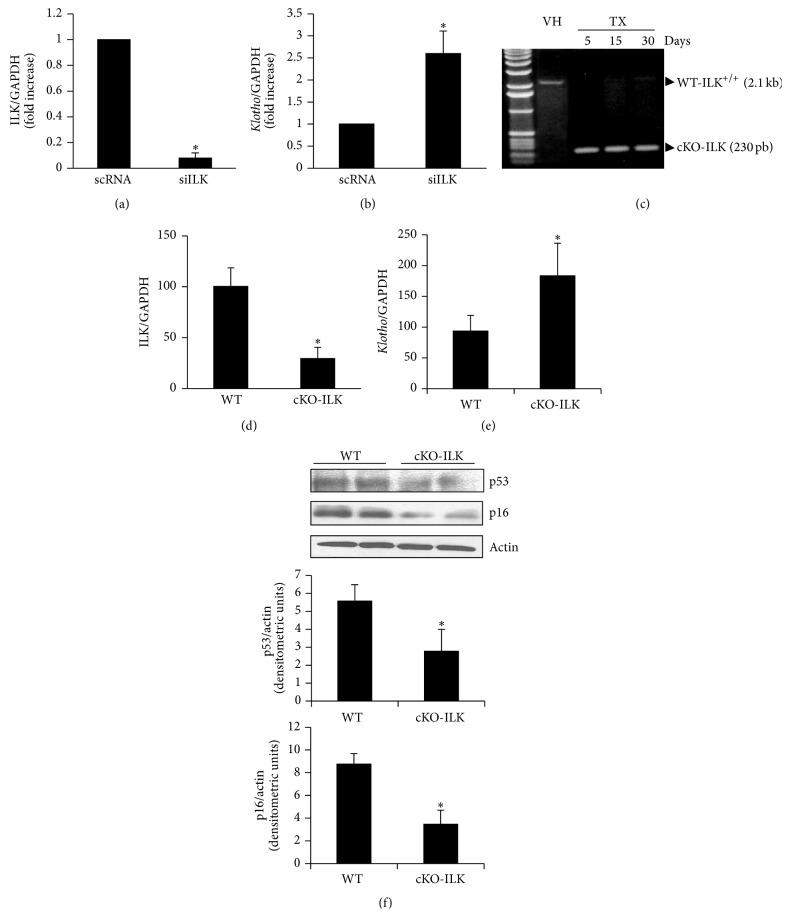
*Increased ILK expression by GOx induced decrease in Klotho expression*. (a, b) MCT cells were transfected with specific siRNA for ILK (siILK) or unspecific scramble siRNA (scRNA) as control. ILK (a) and* Klotho* (b) mRNA expression was evaluated by RT and real time PCR. Bar graph represents the mean ± s.e.m. of three different experiments, and the results are expressed as densitometric units. (c) A conditional ILK knockout mouse model (cKO-ILK) was used and, after treatment with TX for several days, ILK deletion was confirmed versus VH treatment in those mice by PCR of genomic DNA isolated from kidney. ILK (d) and* Klotho* (e) mRNA expression was analyzed in kidney from WT and cKO-ILK mice by RT and real time PCR. Bar graph represents the mean ± s.e.m. of 10 animals per group. (f) p53 and p16 protein expression was analyzed in kidney from WT and cKO-ILK mice by western blot. A representative blot is shown. Bar graphs represent the densitometric analysis of the bands. The results are expressed as densitometric units and are the mean ± s.e.m. of 10 animals per group. ^*∗*^ *p* < 0.05 versus control (scRNA in panels (a)-(b) and WT in the rest panels).

To analyze whether there was a relationship between the reduction of* Klotho* and the increase in ILK expression, we analyzed* Klotho* expression in MCT cells transfected with specific siRNA against ILK. Cells transfected with siILK showed a stronger reduction in ILK mRNA expression (Figure 5(a)) and a stronger increase in* Klotho* mRNA expression (Figure 5(b)) than cells transfected with the unspecific siRNA (scRNA), which were evaluated by RT-qPCR.

In addition, this effect was confirmed by using a conditional ILK knockout mouse model (cKO-ILK). After treatment with TX for several days, ILK deletion was confirmed by PCR (Figure 5(c)). ILK expression was reduced in renal cortex from cKO-ILK versus WT mice (Figure 5(d)). The reduction in ILK gene expression was parallel with a higher* Klotho* gene expression in cKO-ILK mice versus WT (Figure 5(e)), as we found in MCT cells.

Additionally, the protein content of senescence genes p53 and p16 was analyzed in the renal cortex isolated from cKO-ILK mice, finding lower expression than in WT mice (Figure 5(f)).

### 3.4. HEK293T Cells Showed the Same Response to GOx Treatment of ILK and Senescence

**Figure 6:**
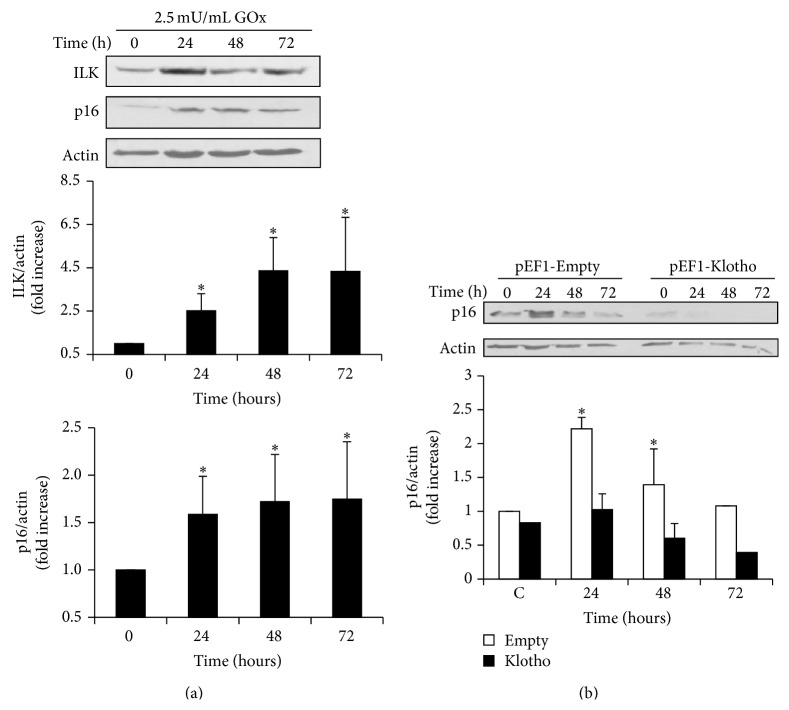
*GOx induced senescence through ILK in other renal cells*. HEK293T cells were treated with 2.5 mU/mL GOx for 24, 48, or 72 h. (a) ILK and p16 protein expressions were analyzed by western blot. A representative blot is shown in each case. Bar graphs represent the densitometric analysis of the bands. The results are expressed as densitometric units and are the mean ± s.e.m. from six different experiments. (b) HEK293T cells were transfected with Klotho protein (pEF1-Klotho, closed bars) or empty vector (pEF1-Empty, open bars) and then treated with 2.5 mU/mL of GOx for 24, 48, and 72 hs. p16 protein expression was evaluated by western blot. A representative blot is shown. Bar graphs represent the densitometric analysis of the bands. The results are expressed as a percentage of control and are the mean ± s.e.m. from five different experiments. ^*∗*^ *p* < 0.05 versus control or time 0.

To assess whether oxidative stress induces senescence in other cultured cells proceeding from kidney and whether the mechanisms involved were similar to those of MCT cells, HEK293T cells were treated with 2.5 mU/mL of GOx for 24, 48, and 72 h. Then, ILK and p16 expressions were analyzed by western blot.  Figure 6(a) shows that GOx induced an increased expression of both ILK and p16 with a similar pattern to the expression found in MCT cells. In addition, HEK293T cells were transfected with* Klotho* expression vector to analyze whether Klotho overexpression was able to inhibit the increase in p16 expression after GOx addition. Results shown in Figure 6(b) demonstrated that cells overexpressing Klotho did not present the expected increase in the senescence gene p16.

## 4. Discussion

The free-radical theory of aging is one of the most popular theories in aging research and the role of oxidative stress in cellular senescence has been extensively studied. Many stressor conditions, such as hyperosmolar stress [9] and advanced glycation end products [10], are able to induce premature senescence in renal cells by disrupting the balance between reactive oxygen species (ROS) generation and the activity of antioxidant systems. However, the mechanisms activated after the manifestation of oxidative stress, which lead to cellular senescence, are less known. For this reason, in this work, we explored some of the mechanisms which could be activated by oxidative stress to lead cells to senescence. To induce a chronic oxidative stress, we used glucose oxidase, as previously described [38, 39]. GOx is an enzyme, absent in mammalian cells [40], which converts oxygen to hydrogen peroxide, in a stoichiometrically simple 1 : 1 relationship, using glucose as substrate. Its product, D-gluconolactone, is metabolically inert [41, 42]. The activity of GOx is stable and remains fully active over 24 h. For this reason, we replaced the medium with fresh medium and GOx every 24 h [40]. In our experiment, GOx addition increases the oxidative damage of proteins in a time-dependent way. Hydrogen peroxide is well known as a tissue damage mediator in several pathological conditions. In the kidney, hydrogen peroxide is known to play an important role in the pathogenesis of ischemia-reperfusion events in tubular cells [43], in CKD [44], and in several inflammatory kidney diseases [45, 46]. Depending on the kidney environmental conditions, oxidative stress generated by hydrogen peroxide induces cell proliferation, apoptosis, and fibrosis [43, 47–49], leading to tissue damage. We hereby propose that a possible mechanism for tissue damage caused by hydrogen peroxide could be the induction of cellular senescence in renal cells. The accumulation of senescent cells in tissues has been identified as a mechanism involved in tissue dysfunction. In this regard, hydrogen peroxide is described to cause senescence in HK-2 tubular cells in a CKD experimental model [50], inducing cell loss. We found that oxidative stress induced by GOx increased senescence in MCT cells in culture 24 h after addition, which were identified to express SA-*β*-GAL activity. Cellular senescence is defined as an irreversible cell cycle arrest characterized by the expression of the cell cycle inhibitor p16 or/and the tumor suppressor p53 [51]. Immortal MCT cells under GOx treatment show an increase in p16 but not in p53 gene expression. The increase in the expression of p16 has been described as a robust biomarker for cellular senescence [52] and has been associated with many age-related diseases [53]. Both increased expression of p16 protein and increased SA-*β*-GAL activity allow us to confirm that prolonged exposition to GOx induced cellular aging in MCT cells.

Next step was to elucidate how oxidative stress leads to senescence. Recently, it has been reported that hydrogen peroxide increases TGF-*β*1 expression through ILK in glomerular mesangial cells [39]. ILK is a component of the intracellular complex that links transmembrane integrins with the actin cytoskeleton and other intracellular signaling pathways [54]. ILK regulates proliferation, differentiation, and motility in many cell types and has recently been involved in cellular senescence [55, 56]. For this reason, we analyzed whether oxidative stress due to hydrogen peroxide also increases ILK expression in MCT cells and found a significant increase after GOx treatment. ILK has a pseudokinase activity [57], which can be measured by the phosphorylation of one of its substrates, GSK-3*β* [58]. Besides, an increase in phospho-GSK-3*β* was found in MCT cells. In order to assess a direct relationship between the increase in ILK expression and activity and cellular senescence, ILK protein was overexpressed by transfecting MCT cells with a plasmid containing the wild-type ILK gene. ILK and p16 expression levels increased 3-fold in transfected cells as compared to nontransfected ones, even in the absence of oxidative stress. Moreover, MCT cells transfected with small interfering RNA against ILK to silence ILK expression did not undergo senescence despite the GOx addition. These results clearly indicate that ILK overexpression has a critical role in cellular senescence induced by oxidative stress. In accordance with this result, it has been described that genetic reduction of ILK in both* C. elegans* and* Drosophila* led to lifespan extension [59].

One of the antiaging factors most studied is the* Klotho* gene, which is expressed mainly in renal tubular cells; Klotho protein protects cells from apoptosis and senescence [60, 61] and from oxidative damage [62]. We analyzed the effect of oxidative damage on the expression of* Klotho* gene in MCT cells and found a reduction in* Klotho* mRNA expression.* Klotho* expression is downregulated in multiple pathological conditions, such as hyperlipidemia, hypertension, and CKD [63, 64]. A significant correlation was found between Klotho levels and oxidative damage in the serum of CKD patients [65]. Our results show that chronic oxidative stress induces a significant reduction in* Klotho* expression, which could lead to senescence, since cells with an ectopic overexpression of Klotho protein did not undergo senescence after GOx addition.

Therefore, we explored whether the reduction of Klotho protein was linked to the increase in ILK under oxidative stressing conditions. For this purpose, we performed two different experimental approaches. First, we downregulated ILK expression by transfection with siILK and found an increase in the transcription of* Klotho* gene. Second, we analyzed the* Klotho* expression in a conditional KO of ILK animal model, as previously reported [37]. Conditional KO-ILK mice showed a strong reduction in renal ILK expression, which was accompanied with an increase in the* Klotho* expression as compared with WT mice. Finally, p16 and p53 senescence gene expression was analyzed in the renal cortex from these animals. Results showed a significant reduction in the expression of both genes.

In accordance with these results, a work performed in* C. elegans* has recently reported that genetic reduction in ILK induced stress response genes related to antioxidant response and heat shock proteins [60]. We hereby propose that the reduction of ILK expression induces* Klotho* gene as a mechanism of stress response. Klotho protein decreases with age [66] and in many age-related diseases [67], but there are few works about the transcriptional regulation of the* Klotho* gene. Among these, it has been described that the hypermethylation of* Klotho* gene promoter increases with age, reducing its expression [68]. Similar results were found in HEK 293T cells, suggesting that ILK plays an important role in renal aging.

## 5. Conclusion

**Figure 7:**
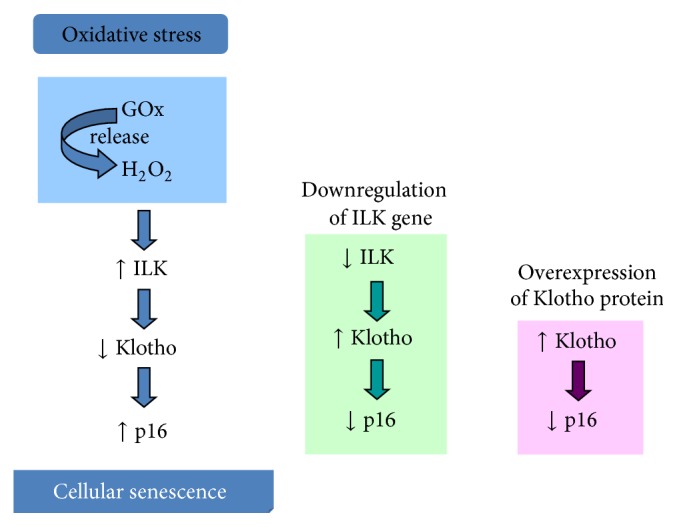
*Proposed integrative mechanisms involved in cellular senescence induced by chronic oxidative stress in renal cells*. Oxidative stress increases ILK activity and expression, which in turn reduces* Klotho* gene expression. Cells with reduced levels of Klotho are less protected from oxidative stress and undergo senescence.

We conclude that chronic oxidative stress induces senescence in renal cells by increasing ILK activity and expression, which in turn reduces* Klotho* gene expression. Cells with reduced levels of Klotho are less protected from oxidative stress and undergo senescence (Figure 7).

Evidence suggesting ILK as a novel downregulator of* Klotho* gene expression is presented here. Further experiments will be necessary to analyze the potential therapeutic implications of the inhibition of ILK in age-related renal pathologies.
