# Corrigendum to “Glucose Oxidase Induces Cellular Senescence in Immortal Renal Cells through ILK by Downregulating *Klotho* Gene Expression”

**DOI:** 10.1155/2016/8392708

**Published:** 2016-05-24

**Authors:** Nuria Troyano-Suárez, María del Nogal-Avila, Inés Mora, Patricia Sosa, Susana López-Ongil, Diego Rodriguez-Puyol, Gemma Olmos, María Piedad Ruíz-Torres

**Affiliations:** ^1^Departamento de Biología de Sistemas, Universidad de Alcalá, Alcalá de Henares, 28871 Madrid, Spain; ^2^University of Alabama at Birmingham, Birmingham, AL, USA; ^3^Unidad de Investigación, Fundación para la Investigación Biomédica del Hospital Universitario Príncipe de Asturias, Alcalá de Henares, 28871 Madrid, Spain; ^4^Instituto Reina Sofía de Investigación Nefrológica, IRSIN, Madrid, Spain

In the article titled “Glucose Oxidase Induces Cellular Senescence in Immortal Renal Cells through ILK by Downregulating* Klotho* Gene Expression” [1] The authors wish to acknowledge the following funding information which was omitted from the above article. This work was supported by Health Institute Carlos III (PI13/02270 and PI13/00336) cofunded with FEDER funds.
